# Vacuum-Assisted Closure Therapy: A Lifesaver in a Case of Neck Necrotizing Fasciitis

**DOI:** 10.7759/cureus.44291

**Published:** 2023-08-28

**Authors:** Saleh A Ba-shammakh, Bourhan M Alrayes, Hamza M Abu-obead, Mohammed A Ba-Shammakh, Fahmi M Al-Mohd, Ebrahim H Ramzoun, Laith Abu-Nowar

**Affiliations:** 1 Department of General Surgery, The Islamic Hospital, Amman, JOR; 2 Department of Oral and Maxillofacial Surgery, University of Aden, Aden, YEM; 3 Department of Oral and Maxillofacial Surgery, The Islamic Hospital, Amman, JOR

**Keywords:** debridement, deep neck infections, negative wound pressure therapy (nwpt), mediastinitis, odontogenic infections, vacuum-assisted closure (vac), necrotizing fasciitis (nf)

## Abstract

Necrotizing fasciitis (NF) is a severe and rare soft tissue infection with a high potential for mortality, particularly in cases related to odontogenic infections in immunocompromised patients. The conventional treatment for NF includes broad-spectrum antibiotics and aggressive surgical debridement. This report presents a unique case of a 34-year-old healthy male who developed NF following a lower left wisdom tooth extraction. The infection extended into the superior mediastinum, requiring emergency surgical intervention. The therapeutic management included vacuum-assisted closure (VAC), a treatment modality showing promise in managing complex soft tissue infections, in combination with other adjunct treatments. The patient showed a satisfactory healing process and no signs of recurrence during the six-month follow-up period. This case underlines the importance of early diagnosis and the potential benefit of VAC therapy in managing advanced NF, emphasizing the need for further research and clinical application.

## Introduction

Necrotizing fasciitis (NF), although uncommon, presents a potentially lethal threat in the form of a soft tissue infection, leading to widespread necrosis and damage to the skin and the tissue lying underneath the skin [1]. As far back as the 5th century BC, Hippocrates became the first to identify this horrifying ailment, which is notable for its rapid and severe progression, eventually resulting in a comprehensive necrosis that affects both the superficial fascial layer and related cutaneous tissue [2].Although its occurrence is infrequent, instances linked to odontogenic infections are noticeable, with the compromised immune system being a considerable risk factor [3-4]. The swift spread of this infection to nearby tissues may result in significant tissue loss, mediastinitis, multiple organ failure, or even mortality, emphasizing the urgency for prompt diagnosis and surgical intervention [3,5].

Vacuum-assisted closure (VAC), a treatment modality first brought into use by Argenta and Morykwas in 1997, has exhibited effectiveness in handling intricate soft tissue infections, such as NF [6]. The method of treatment utilizes negative pressure, conveyed through a lattice-like foam enveloped in a sticky drape, which advances patient care through several mechanisms. These include the drainage of fluid buildup or exudate, augmentation of blood flow within the wound vicinity, and stimulation of cellular expansion in the granulation tissue as a preparatory measure [6-7]. However, its usage in addressing NF, especially in the head and neck region, remains insufficiently recorded despite the anticipated benefits [8].

VAC therapy may confront challenges, such as the presence of a malignant wound, unaddressed osteomyelitis, non-enteric and unexplored fistulas, necrotic tissue marked by eschar, and susceptibility to silver, among other considerations [7]. Despite these concerns, VAC therapy, when used in conjunction with adjuncts, such as GELFOAM®, and antimicrobial barrier dressings, such as ACTICOAT®, could substantially enhance patient prognosis in NF scenarios, particularly in instances of severe deep neck infections [9-13]. Nonetheless, the technical complexity and potential hazard of accessing vital organs during surgical procedures should not be disregarded [14-15]. Herein, we present a successfully managed case of NF following a dental extraction, showcasing the potential effectiveness of VAC therapy. To validate these results, additional investigations and clinical implementation are warranted.

## Case presentation

A 34-year-old previously healthy male came to our hospital through the emergency department complaining of neck swelling that started one week following a lower left wisdom tooth extraction. The patient initially noticed a small, painless swelling in his neck, which grew in size over a week, becoming increasingly painful, characterized first by a dull ache that progressed to a throbbing sensation. Accompanying this was an increasing difficulty and discomfort in swallowing, compounded by recurrent bouts of fever.

Over the following days, the patient sought medical advice twice. Initially, he was prescribed oral antibiotics and analgesics by his general physician, but his symptoms remained unimproved. Following a second consultation, the patient was given additional antibiotics and a course of steroids, which proved ineffective. Instead, his condition worsened, with the swelling spreading to encompass the entirety of his neck and upper chest. Moreover, he reported exacerbated difficulty in swallowing and breathing.

Upon presentation, the patient was fully conscious, alert, and oriented. His vital signs were as follows: temperature 37.9 °C, heart rate (HR) 85 BPM, blood pressure (BP) 145/79 mmHg, and oxygen saturation (O_2_ sat) 94%. There was a notable exception of a markedly elevated C-reactive protein (CRP) of 277 and a white blood cell count of 14.5. His physical examination was generally unremarkable, except for a noticeable bilateral neck swelling that was tender, warm to touch, and well-demarcated with erythema extending to the xiphoid process and accompanied by a deviation of the trachea.

**Figure 1:**
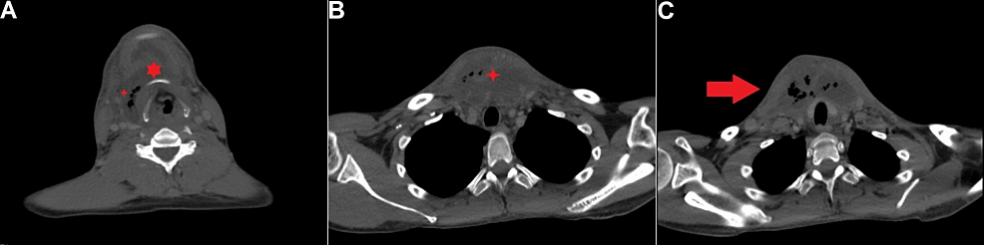
Neck and chest computed tomography (CT) scan A: Neck CT scan at the level of C4, showing a bilateral submandibular and parapharyngeal neck abscess, more on the right side, with gas foci causing obliteration of the right parapharyngeal pad and deviation of the larynx to the left side. B: Chest CT scan, showing anterior mediastinal fluid collection with air foci and bulge of overlying skin, indicating descending mediastinitis. C: Neck CT scan at the level of the thoracic inlet, showing an anterior mediastinal fluid collection with gas foci and bulging of the overlying skin, indicating an inflammatory process.

A computed tomography (CT) scan of the neck showed an extensive collection in the right submandibular, submental, central, and right side of the neck areas, crossing the midline to the left side of the neck and extending into the superior mediastinum and retropharyngeal space, indicating a multi-compartment neck space abscess extending into the superior mediastinum (Figure 1).

To secure his airway, the patient was electively intubated upon admission and started on clindamycin and piperacillin/tazobactam regimen.

**Figure 2:**
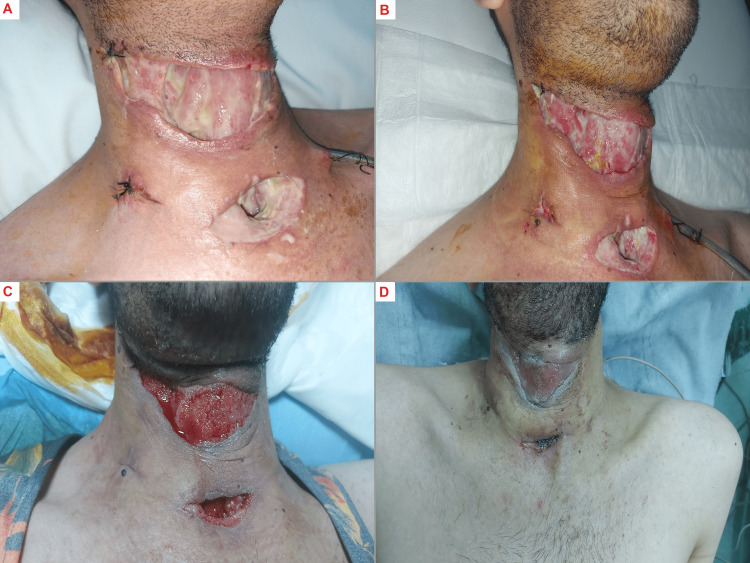
Wound healing progression with vacuum-assisted closure (VAC) dressing A: Postoperative day 2 after bedside dressing, showing the initial stages of the healing process. B: Postoperative day 5 after bedside dressing, highlighting further progression in wound recovery. C: Wound after one week of VAC dressing, illustrating continued improvement and wound care. D: Wound after two weeks of VAC dressing, representing further healing and the patient's readiness for the next stage of treatment.

The following day, the patient underwent incision, drainage, and mediastinoscopy to remove the pus collection. Approximately 300 ml of pus were suctioned, and two VacDrain tubes (ACTIV.A.C.™ Therapy System, 3M+KCL, United States) were placed on his neck's right and left sides (Figure 2).

**Figure 3:**
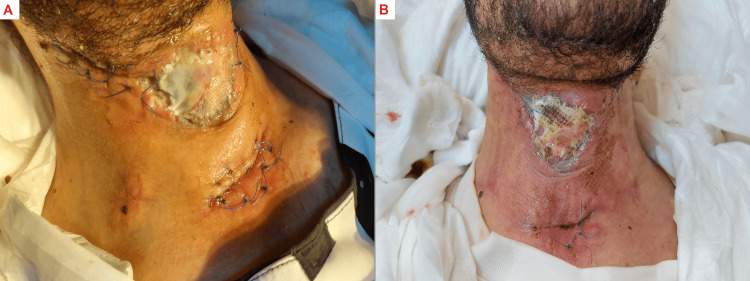
Skin grafting A: Image taken one week after the skin grafting in the outpatient clinic (OPC), showing the early stages of healing. B: Image taken two weeks after the skin grafting in the OPC.

Postoperatively, the patient was admitted to the intensive care unit (ICU), where the pus was sent for culture, which later returned negative results. The patient remained generally well during his ICU stay, with no fever spikes, and his vital signs remained stable. The patient's treatment regimen included piperacillin/tazobactam with clindamycin while admitted intravenously then discharged continued on oral amoxicillin/clavulanate and clindamycin. Surgical neck exploration was performed next morning, and then two days later, he underwent pus drainage and debridement, followed by comprehensive bedside dressings daily. On the third day, after the operation, the patient was extubated. The patient's hospital stay was extended to two weeks, after which a skin graft was applied (Figure 3).

The patient remained in the hospital for another two days following the skin grafting procedure, culminating in a total hospital stay of 16 days before discharge.

**Figure 4:**
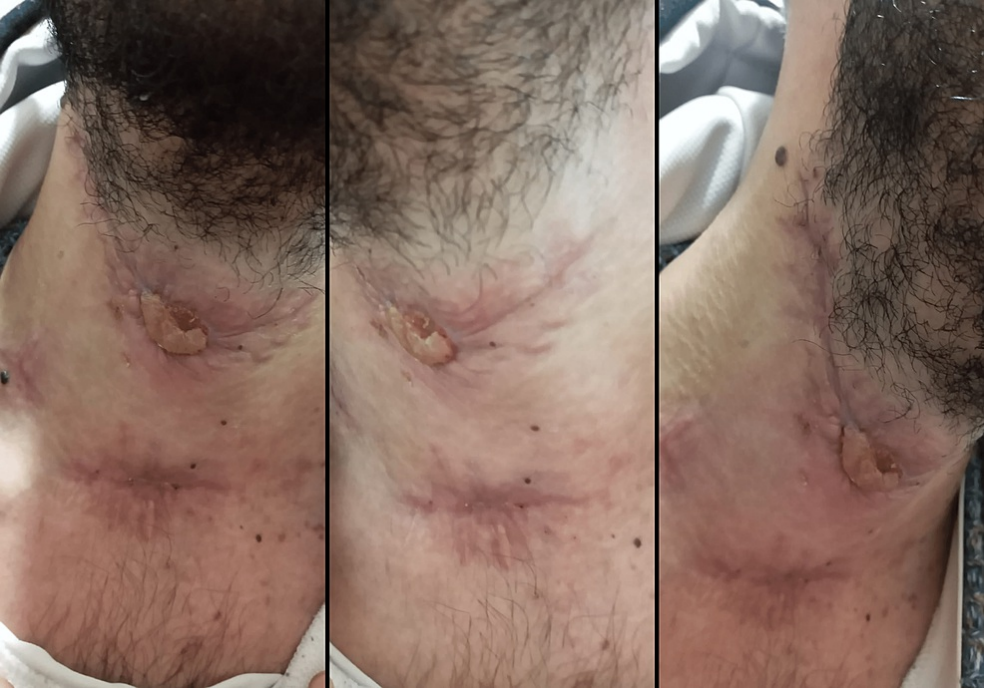
Successful recovery at the follow-up appointment Image of the patient's neck during a follow-up appointment, showing satisfactory healing and indicating successful recovery without signs of recurrence or complications.

A detailed follow-up plan was established for the patient post-discharge to monitor the healing process and check for any signs of recurrence or complications. Regular follow-up appointments were scheduled at the one-month and three-month marks. The patient's general health status, the healing process, and potential complications were closely monitored during these visits. Each routine appointment confirmed satisfactory healing and no signs of recurrence, indicating a successful recovery (Figure 4).

## Discussion

NF represents an aggressive type of necrotizing soft tissue infections (NSTIs), which rapidly evolves, marked by pus-filled tissue disintegration that can potentially affect structures, such as the skeletal muscle, fascia, epidermis, dermis, and subcutaneous fat [16]. A relatively rare occurrence of this condition is cervicofacial necrotizing fasciitis (CNF), observed in the head and neck region. Its rarity is primarily due to the region's rich vascular network, which expedites the immune response to combat infections efficiently [17]. Dental infections and surgical procedures are frequently linked to CNF. While generally sparing immunocompetent individuals and those without significant medical history, this condition predominantly affects individuals with chronic diseases, such as diabetes mellitus, obesity, alcoholism, and peripheral vascular disease [18-19].

The rapid advancement of CNF manifestations calls for prompt identification and an inclusive therapeutic strategy encompassing hemodynamic assistance, wide-ranging antibiotics, and surgical debridement [20]. Odontogenic infections often lead to NF [19], and the infection can quickly extend along the fascial planes into the neck or chest, possibly leading to serious conditions, such as mediastinitis, thereby elevating the comorbidity risk to about 50% [21]. Prompt and assertive medical and surgical interventions are vital for successful patient outcomes [22].

Frequently, the root causes of odontogenic cervical NF are infections of the second and third mandibular molars [23]. Infections in these regions can readily propagate to the submandibular space, infiltrate the adjacent areas, and eventually extend to the skull base or down into the mediastinum and thoracic cavity [24]. Infections stemming from these teeth have the potential for rapid diffusion, involving all fascial layers of the head and neck.

Effectively treating NF, particularly when intertwined with severe deep neck infections, demands a vigorous strategy that includes safeguarding the airway, correctly applying antibiotic treatments, and conducting surgical operations to drain abscesses [12]. The necessity for surgical intervention can be determined by several factors, such as compromised airway, presence of sepsis, descending infection , conditions like diabetes mellitus, or a lack of observable progress within two days of commencing intravenous antibiotic therapy [25-26]. In addition, when abscesses reach or exceed 3 cm in size or influence the prevertebral, anterior visceral, or carotid spaces, surgical drainage becomes imperative [13].

Negative wound pressure therapy (NWPT) has demonstrated multiple benefits compared to conventional treatment modalities. NWPT, a process that aids wound healing via application of regulated sub-atmospheric pressure [27], necessitates fewer dressing changes, mitigates local edema, encourages perfusion and granulation tissue development, and enhances the availability of growth factors and enzymes essential for epithelial surface regeneration [28]. Moreover, it spurs angiogenesis and arterial dilation around the wound, thereby delivering oxygen at high concentrations [28]. In tandem with antibiotic therapy, NWPT can transform the wound micro-environment, suppressing anaerobic bacterial proliferation and mitigating the onset of new infections [29].

While the guidelines for NWPT use in head and neck applications remain ambiguous, there have been instances where it has proved efficacious in treating NF. Remarkably, it was advantageous in a case of extensive NF in the head and neck devoid of associated diseases, obviating the necessity for reconstructive surgery. The use of negative pressure systems, such as NWPT, is suggested to outperform traditional deep neck treatments, even for severe infections extending into the mediastinal region [30].

This case report, while highlighting the effective use of VAC therapy for neck NF, presents several limitations. First, it represents a single patient's experience and response to VAC therapy. Therefore, its generalizability is limited due to potential variations in individual patient characteristics and underlying health conditions. It should be noted that similar results cannot be assured in all comparable cases. Second, there needs to be more wide-ranging literature documenting the use of VAC therapy for neck NF. Further extensive research is needed to affirm this therapy's efficacy, safety, and procedural best practices. Despite these limitations, our case report offers an important addition to the field by demonstrating a novel treatment approach for neck NF.

## Conclusions

NF, particularly in the head and neck region, can pose a significant health threat, warranting early diagnosis and prompt aggressive treatment. This case demonstrates that VAC, despite its limited documentation in NF management, can contribute to positive patient outcomes when combined with other treatment modalities, even in advanced cases of deep neck infections. However, surgical treatment's risks and technical challenges in proximity to vital organs necessitate careful consideration. This case prompts a re-evaluation of the established treatment regimens and encourages further exploration and research into the potential benefits of VAC therapy in managing NF. Ultimately, this may improve patient outcomes and reduce associated morbidity and mortality rates.
